# Predialysis therapeutic care and health-related quality of life at dialysis onset (The pharmacoepidemiologic AVENIR study)

**DOI:** 10.1186/1477-7525-9-7

**Published:** 2011-01-24

**Authors:** Stephanie Boini, Luc Frimat, Michele Kessler, Serge Briançon, Nathalie Thilly

**Affiliations:** 1 Clinical Epidemiology and Evaluation, CIC-EC CIE6 Inserm, University hospital of Nancy, France; 2 Nancy University, P. Verlaine - Metz University, Paris - Descartes University, EA 4360 Apemac, Nancy, France; 3 Nephrology, University hospital of Nancy, France

## Abstract

**Background:**

To determine the impact of the quality of pre-dialysis nephrological care on health-related quality of life (HRQoL) at dialysis onset, which has not been well evaluated.

**Methods:**

All adults who began a dialysis treatment in the administrative region of Lorraine (France) in 2005 or 2006, were enrolled in this prospective observational study.

HRQoL was measured using the Kidney Disease Quality of Life V36 questionnaire, which enables calculation of two generic (physical and mental) and three specific dimensions (Symptoms/problems, Effects and Burden of kidney disease). The specific dimensions were scored from 0 to 100 (worst to best possible functioning). Pre-dialysis nephrological care was measured using three indicators: quality of therapeutic practices (evaluated across five main aspects: hypertension/proteinuria, anemia, bone disease, metabolic acidosis and dyslipidemia), time since referral to a nephrologist and number of nephrology consultations in the year preceding dialysis treatment.

**Results:**

Two thousand and eighty-three (67.4%) patients were referred to a nephrologist more than 1 month before dialysis initiation and completed the HRQoL questionnaire. Quality of therapeutic practices was significantly associated with the Mental component. Time since referral to a nephrologist was associated with Symptoms/problems and the Effects of kidney disease dimensions, but no relationship was found between the number of nephrology consultations and HRQoL.

**Conclusions:**

HRQoL at dialysis onset is significantly influenced by the quality of pre-dialysis nephrological care. Therefore, disease management should be emphasized.

## Background

Although the correlation between chronic kidney disease (CKD) and risk of cardiovascular morbidity and mortality has been thoroughly investigated, studies evaluating the impact of CKD on health-related quality of life (HRQOL) are somewhat scarce [1-3]. In particular, the relationship between quality of pre-dialysis care and HRQoL at dialysis onset has not been investigated to date. However, numerous studies have shown associations between quality of pre-dialysis care and dialysis mortality on one hand [4] and, HRQoL at dialysis onset and dialysis mortality on the other hand [5-7].

The quality of pre-dialysis care is a multidimensional concept that includes several aspects, for example, clinical follow-up by nephrologists, the quality of therapeutic care, the quality of dialysis preparation, and counselling.

A positive association between early referral to a nephrologist and survival after starting renal replacement therapy (RRT) has been clearly demonstrated [8] but the impact of early referral on HRQoL at initiation of dialysis is still a matter for debate [2,9]. Moreover, the lack of a consensus over the definition of 'early' and 'late' nephrology referral has left primary care providers unsure about the optimum timing and pattern of nephrology care. Nephrological care was recently assessed from a quantitative rather than a qualitative perspective, focusing on the number of nephrology consultations before RRT [10]. Moreover, a favourable association between early referral or a high number of pre-ESRD nephrology consultations and quality of therapeutic care has been suggested [11,12]. Likewise, quality of pre-ESRD therapeutic practices has been found to be associated with survival after RRT [4].

We used data from the pharmacoepidemiologic AVENIR (AVantagE de la Néphroprotection dans l'Insuffisance Rénale) study to explore the impact on HRQoL at dialysis onset of three pre-dialysis indicators of quality of care: quality of therapeutic practices, time since referral to a nephrologist and number of nephrology consultations during the year preceding dialysis. Our hypothesis is that the higher the quality of pre-dialysis care, the better the HRQoL. Our aim is to heighten nephrologists' awareness of the outstanding importance of the quality of pre-dialysis care.

## Methods

### Setting, study design and sample selection

The AVENIR study was an observational cohort study involving 12 private and public nephrology units operating in the administrative region of Lorraine, northeast France (population of 2,339,000, according to the 2006 census). Its methodology was approved by the ethics committee of the regional university hospital and is described in detail elsewhere [11].

All adults with CKD who began a dialysis treatment in one of the 12 units between January 1, 2005, and December 31, 2006, were identified from the regional ESRD registry (REIN registry) and enrolled in the AVENIR study. Patients with reversible renal failure and those returning to dialysis following kidney graft failure were not included. The present analysis focuses on the impact of several features of pre-dialysis nephrological care on HRQoL of ESRD patients referred to a nephrologist at least 1 month before the start of dialysis.

### Data collection and definitions

A standardized form was used to retrospectively collect demographic, clinical, biological and therapeutic data from outpatient medical records. Demographic and clinical data (except for blood pressure) were from inclusion in the REIN registry. Blood pressure readings, as well as biological and therapeutic data covered the observation period from the day of the first nephrology consultation to dialysis onset, and were used to evaluate the quality of therapeutic practices. Demographic and clinical variables used as adjustment factors in the analysis included age, gender, body mass index (BMI), primary renal disease and the presence (or absence) of at least one co-morbidity. BMI was calculated as weight (kg)/square of height (m). Primary renal disease was categorized into five groups: glomerulonephritis, diabetic or hypertensive nephropathy, hereditary nephropathy and others. Co-morbidity was defined as the presence of clinically significant non-renal disease (e.g. cardiac disease, vascular disease, respiratory disease, diabetes mellitus and malignancy).

In addition, all patients who began a dialysis treatment had to complete a HRQoL questionnaire as soon as possible after their first session, and within the first 3 months of replacement therapy.

### Quality of therapeutic practices

The appropriateness of pre-dialysis therapeutic practices was assessed in terms of adherence to current guidelines [13-17] covering five main aspects of therapeutic care in CKD: hypertension/proteinuria, anemia, bone disease, metabolic acidosis and dyslipidemia. A practice was considered inappropriate if one treatment was not prescribed when it was indicated for a biological or clinical reason; otherwise, the practice was considered appropriate (Table 1). For example, hypertensive care was recorded as inappropriate for a patient not given antihypertensive medication when his or her mean blood pressure during the observation period was >130/80 mmHg. More detailed information has been published elsewhere [11].

**Table 1:** Definition of 'Inappropriate therapeutic care' and percentage of patients being managed appropriately by therapeutic aspect evaluated (n = 420 included) [10]

Therapeutic fields evaluated	Definition of 'Inappropriate therapeutic care'	% of patients being managed appropriately
Hypertension/Proteinuria	Mean BP^a ^>130/80 mmHg without prescription of an antihypertensive agent	72.4
	Mean proteinuria >0.5 g/dl without prescription of a renin-angiotensin system inhibitor	
Anemia	Hemoglobin <11 g/dl in two successive readings without prescription of an erythropoiesis-stimulating agent	
	Erythropoiesis-stimulating therapy without prescription of iron	56.2
	Or	
	Mean serum ferritin <100 ng/ml without prescription of iron (in patients not given erythropoiesis-stimulating therapy)	
Bone disease	Mean serum calcium <10.2 mg/dl without prescription of calcium	16.7
	Mean serum 25-hydroxyvitamin D <30 ng/ml without prescription of ergocalciferol	
	Or	
	Mean serum 25-hydroxyvitamin D >30 ng/ml and hyperparathyroidism without prescription of alfacalcidol	
Metabolic acidosis	Mean serum bicarbonates <23 mEq/l without prescription of bicarbonate	60.2
Dyslipidemia	Mean fasting total cholesterol >201 mg/dl or mean triglycerides >150.5 mg/dl without prescription of a lipid-lowering therapy	61.4

^a ^BP, blood pressure.

The quality of therapeutic practices was then estimated for each patient in terms of the number of aspects (out of the five above) being managed appropriately. Quality of practices was considered to be High when four or five aspects were appropriately managed, Moderate when including two or three aspects and finally Poor when none or just one aspect was appropriately managed.

### Pre-dialysis nephrology care

Pre-dialysis nephrology care was assessed in terms of the timing of referral to a nephrologist before dialysis onset and the number of nephrology consultations during the year preceding dialysis treatment.

Patients were classified into three groups according to their timing of referral to a nephrologist as follows: more than 12 months before dialysis onset (early referral), less than 12 months and more than 4 months (intermediate referral), and less than 4 months and more than 1 month (late referral). The number of nephrology consultations during the year preceding dialysis was categorized into three groups: 0 to 2 consultations, 3 to 5 consultations, and 6 consultations or more.

### Outcome of interest

HRQoL was measured with the French version of the 'Kidney Disease Quality of Life' (KDQoL) V36 questionnaire [18]. This instrument includes a 12-item health survey as the generic core (SF12), supplemented with multi-item scales targeted at particular concerns of patients with kidney disease and on dialysis.

The 12 items of SF12 - a shorter version of the generic SF36 instrument - may be combined into two summary measures: Physical (PCS12) and Mental (MCS12) Component Summary Scales [19]. They are computed to have means of 50 and standard deviations of 10 in a general US population. The specific items may be summarized into three dimensions: symptoms/problems (12 items), effects of kidney disease on daily life (8 items), and burden of kidney disease (4 items) [20]. All these specific dimensions, scored from 0 to 100 (worst to best possible functioning), are calculated as the mean of item values when no more than half of the items are missing. Otherwise, scores are recorded as missing.

We calculated the Cronbach coefficient of the three specific dimensions, confirming their internal consistency in our sample (0.76, 0.77 and 0.79 for Symptoms, Effects and Burden dimensions, respectively).

### Statistical Analysis

Descriptive statistics were used to assess patients' characteristics according to whether or not they had completed the KDQoL questionnaire (respondents/non-respondents). Continuous variables are presented as means ± standard deviations and categorical variables as percentages. Comparisons between respondents and non-respondents were made using the Pearson Chi^2 ^test and analysis of variance for categorical and continuous variables, respectively.

Analysis of variance models were used to explore the impact of the three pre-dialysis indicators defined above on each HRQoL score at dialysis onset in a bivariable analysis. Indicators significantly associated with HRQoL in the bivariable analysis were then candidates in a multivariable analysis of variance model, adjusted for the main patient characteristics known to be associated with HRQoL in CKD (age, gender, BMI, primary renal disease, co-morbidity) [21-24] and the nephrology unit. The HRQoL scores are reported as means ± standard errors and *P*-value. A *P*-value of < 0.05 for two-sided tests was considered significant. All analyses were performed with SAS version 9.1 (SAS Institute, Inc., Cary, N.C).

## Results

### Patient characteristics

On the 566 patients enrolled in the AVENIR study, 420 were referred to a nephrologist more than 1 month before dialysis initiation and are considered here. Among them, 137 did not complete the KDQoL questionnaire at all (n = 99) or completed it after the third month of dialysis treatment (n = 38). Thus, 283 patients completed the KDQoL questionnaire as indicated and were considered as respondents (response rate= 67.4%).

Table 2 shows the characteristics of included patients overall (n = 420) and by respondent status. Among respondent patients, the mean age was 67.1 ± 14.6 years, and 63.3% were male. Hypertension and diabetes were the leading causes of CKD, and 44.2% of respondents had at least one co-morbidity. The average length of pre-dialysis nephrological care was 43.0 ± 51.9 months, and nearly half of these patients received between 3 and 5 nephrology consultations during the year preceding dialysis.

**Table 2:** Characteristics of included patients according to their respondent status

	Overall (N = 420)	Respondents		
		
		YES (N = 283)	NO (N = 137)	* P*
Male sex (%)	61.0	63.3	56.2	0.17
				
Age at dialysis onset, year				
m ± SD	68.2 ± 14.8	67.1 ± 14.6	70.5 ± 15.1	0.03
<45 (%)	8.6	9.2	7.3	0.06
45 - 64 (%)	23.8	26.9	17.5	
≥65 (%)	67.6	64.0	75.2	
				
Body mass index ≥ 25 kg/m^2 ^(%)	59.8	60.8	57.7	0.55
				
Primary renal disease (%)				
Glomerulonephritis	10.3	11.3	8.1	0.55
Diabetic nephropathy	22.7	20.5	27.2	
Hypertensive nephropathy	23.6	24.4	22.1	
Hereditary nephropathy	5.5	5.7	5.7	
Others	37.9	38.2	37.5	
				
Comorbid condition (%)	47.1	44.2	53.3	0.08
				
Quality of therapeutic practices (%)				
High	22.1	23.7	19.0	0.12
Moderate	65.7	62.5	72.3	
Poor	12.1	13.8	8.8	
				
Time since referral to a nephrologist, months				
m ± SD	42.0 ± 52.3	43.0 ± 51.9	39.9 ± 53.2	0.57
>12 (%)	69.3	72.8	62.0	0.07
[4 - 12[ (%)	17.9	16.3	21.2	
[1 - 4[ (%)	12.9	11.0	16.8	
				
Number of nephrology consultations (%)				
6 or more	24.6	28.0	17.5	0.06
3 - 5	49.2	47.2	53.3	
0 - 2	26.3	24.8	29.2	

As compared with non-respondents, respondents were younger (*P *= 0.03). They also tended to have more pre-dialysis nephrology consultations and were more likely to be referred early to a nephrologist than non-respondents, but these differences did not reach significance.

### HRQoL results

Table 3 shows that HRQoL measured by the SF12 was altered in its physical (PCS12) and mental (MCS12) components: respectively -10.5 and -7.1 points, as compared with the general US population and -10.8 and -4.3 points, as compared with the general French population [25]. The specific scores varied from 41.1 points for the dimension 'Burden of kidney disease' to 67.9 points for 'Symptoms/problems'.

**Table 3:** HRQoL scores at dialysis initiation (N = 283 respondents)

HRQoL scores	N	Mean	Standard Error
Physical (PCS12)	248	39.5	5.8
Mental (MCS12)	248	42.9	7.0
Symptoms/problems	278	67.9	16.8
Effects of kidney disease	280	61.2	20.3
Burden of kidney disease	278	41.1	23.6

Abbreviations: HRQoL, health related quality of life; PCS, physical component summary; MCS, mental component summary.

### Impact of quality of therapeutic practices and pre-dialysis nephrology care on HRQoL

Table 4 presents HRQoL scores for pre-dialysis indicators that were significantly associated with HRQoL dimensions in the multivariable analysis. The Physical Component was influenced by none of the three pre-dialysis indicators. Quality of therapeutic practices was significantly associated with the Mental Component: the higher the quality of practices, the better the MCS12 score (High quality *vs. *Poor = +3.8 points, *P *= 0.01). Time since referral to a nephrologist was associated with two specific dimensions: 'Symptoms/problems' and 'Effects of kidney disease'. The longer the pre-dialysis nephrological follow-up, the better the score related to 'Symptoms/problems' (>12 months *vs. *1 to 4 months = +10.9 points, *P *= 0.001, and 4-12 months vs. 1 to 4 months = +10.5 points, P = 0.007) and the better the score of 'Effects of kidney disease' (>12 months *vs. *1 to 4 months = +8.4 points, *P *= 0.03). The number of nephrology consultations during the year preceding dialysis was associated with none of the five dimensions of HRQoL.

**Table 4:** Impact of quality of therapeutic practices and pre-dialysis nephrology care on HRQoL (N = 283 respondents)

	PCS12			MCS12			Symptoms/problems			Effects of kidney disease			Burden of kidney disease		
					
	Mean*	SE	*P*	Mean*	SE	*P*	Mean*	SE	*P*	Mean*	SE	*P*	Mean*	SE	*P*
**Quality of therapeutic practices**	0.006	0.006	NS			0.05			NS			NS			NS
High				44.6	1.0	0.01									
Moderate				42.8	0.7	0.13									
Poor				40.8	1.3	ref.									
															
**Time since referral to a nephrologist**			NS			NS			0.004			0.09			NS
>12 months							69.9	1.5	0.001	63.5	1.8	0.03			
[4 - 12[							69.5	2.7	0.007	62.9	3.2	0.09			
[1 - 4 [							59.0	3.2	ref.	55.1	3.9	ref.			
															
**Number of nephrology consultations**			NS			NS			NS			NS			NS
6 or more															
3 - 5															
0 - 2															

Abbreviations: HRQoL, health related quality of life; PCS, physical component summary; MCS, mental component summary; SE, standard error; NS, not significant.

*Model adjusted for age, gender, BMI, primary renal disease, presence of co-morbidity, and nephrology unit.

When limiting the analyses to subjects who completed the HRQoL questionnaire within 30 days after dialysis onset (n = 211), all the previously observed associations remained statistically significant. Results remained unchanged too when analyses were re-run with only subjects who completed the questionnaire within the first 10 days after dialysis onset (n = 120).

## Discussion

To our knowledge, this observational study is the first to explore in depth the association between the quality of pre-ESRD nephrological care, evaluated across three indicators, and HRQoL at dialysis onset. In a field where randomized controlled studies cannot be ethically designed, our results suggest: first, a mild, but statistically significant, association between quality of therapeutic practices, evaluated across five therapeutic aspects, and mental, but not physical, quality of life; second, the earlier the referral to a nephrologist, the better the control of symptoms, problems and effects of CKD; third, a lack of association between the number of nephrology consultations and HRQoL.

HRQoL was measured with the validated French version of the KDQoL V36 [20]. The two generic scores allowed ESRD patients to be compared with the general population, whereas the three specific scores explored the impact of the kidney disease on daily life. Both physical and mental summary scores were well below 50, which is the expected average from the US general population. These results are consistent with previous studies [1,7]. Moreover, HRQoL at dialysis onset was altered compared to the French general population, particularly the physical component [25]. Disease-specific scores observed in our ESRD sample were close to scores reported by Molsted *et al *[26], but well below than those observed by Mujais *et al *[27] in their CKD stage V patients. Nevertheless, in this last study, HRQoL was measured when patients were not yet under dialysis treatment, which seems to have a marked impact on the specific dimensions of HRQoL, particularly the 'Burden of kidney disease'. In any case, this dimension was always the most impaired HRQoL dimension in CKD or ESRD patients. This emphasises the need for psychological support of ESRD patients at dialysis onset.

Previous studies using the SF36 suggest that scores in the range of 2 or 3 points on the physical and mental summary scores (equivalent to 0.2 to 0.3 SD) are likely to be clinically important [28]. We observed, in the MCS12 score, differences according to the level of quality of therapeutic practices that were around 2 to 4 points, suggesting that they are likely to be noticeable and meaningful to patients at dialysis initiation. As perceived mental health is an independent predictor of mortality and morbidity [7], attaining the target of MCS12 score observed in patients with high quality of therapeutic practices is of interest. No other study to date has investigated the impact of the quality of pre-ESRD therapeutic practices on HRQoL at dialysis initiation.

Concerning the impact of time since referral to a nephrologist on HRQoL, results from previous studies are conflicting [1,9]. Sesso and Yoshihiro [9] have demonstrated that patients referred late to a nephrologist (≤ one month before starting dialysis) have significantly worse HRQoL than those referred early (≥ 6 months). We found similar tendencies in two of the three specific HRQoL dimensions (symptoms and effects) but no comparison can be made in HRQoL scores because Sesso and Yoshihiro used another quality of life questionnaire (Kidney Disease Questionnaire). These associations we found between time referral to a nephrologist and specific dimensions of HRQoL may reflect the benefit of the nephrologist's having had more time to evaluate and treat properly somatic symptoms and consequences of CKD on his patients. Conversely, Caskey *et al *found no significant difference between early and late referral patients in any of the SF36 summary scores or domain scores [1]. However, in this study, patients were considered 'early referred' if they had been followed by a nephrologist for >1 month before their first dialysis. As we considered early referred patients those who had been followed for more than 12 months before dialysis onset, the results are not comparable. The main difficulty in comparing results of studies investigating the time of referral to a nephrologist is the use of multiple definitions of 'early' and 'late'.

Another way of assessing pre-ESRD nephrological care is to consider the frequency of nephrology consultations before RRT rather than timing of referral [10]. In our study, we found no association between the number of nephrology consultations during the year preceding RRT and HRQoL at initiation of dialysis. Studies published to date have investigated the relationship between the frequency of patient-nephrologist visits during maintenance dialysis and HRQoL [10,29] but none has looked at the impact on HRQoL of frequency of visits before RRT. Moreover, neither Plantinga *et al *[10] nor Mentari *et al *[29] found any association between the frequency of patient-nephrologist contact and HRQoL of dialyzed patients.

Some possible limitations should be considered when interpreting our findings. First, as this study was observational, it allows us to measure associations between pre-dialysis indicators and HRQoL, but cannot demonstrate strictly causal relationships. However, given that a controlled trial in which patients would be randomized on quality of pre-dialysis care is clearly impractical for ethical reasons, our study is of value. Second, almost one third of included patients did not complete the HRQoL questionnaire. Nevertheless, given the relatively minor differences (only age) between respondents and non-respondents, we can assume that these non-responses probably did not introduce a systematic bias that would distort our conclusions. Third, HRQoL was measured up to three months after the start of dialysis. This may reflect care received on dialysis as much as pre-dialysis care, but sensitivity analyses including only patients who completed the HRQoL within the first 10 days after the dialysis onset did not change the results. Fourth, despite adjustment for the main patient characteristics known to be associated with HRQoL in CKD, residual confounding due to the lack of data on variables - such as socioeconomics parameters - for which we could not account may still exist. Fifth, several aspects of quality of pre-dialysis care were taken into account in our study, but not all. For example, the quality of dialysis preparation and counseling was not considered here.

Concerning management of CKD, clinicians have always recognized the importance of diagnosing functional impairments. Our study provides finally an accurate measure of patient-perceived health status at dialysis onset, and highlights the impact of quality of therapeutic practices and early nephrology referral on HRQoL, independently of the number of consultations.

## Conclusions

To our knowledge, this observational study is the first to explore the association between the quality of pre-ESRD nephrological care and HRQoL at dialysis onset. The mental component, but not the physical, is significantly influenced by the quality of pre-dialysis nephrological care, evaluated across five therapeutic aspects. Late referral to a nephrologist is associated with poor HRQoL (symptoms/problems and effects of disease dimensions). Therefore, CKD disease management incorporating psychological support should be emphasized.

## Competing interests

The authors declare that they have no competing interests.

## Authors' contributions

SBo & NT participated in the design of this ancillary work, reviewed the literature, performed the statistical analysis, and drafted the manuscript. LF participated in the design of this work and provided feedback on it. MK and SBr participated in the design and provided feedback. All authors collaborated interactively, and read and approved the final version.
